# Genetic counseling for congenital disorders of glycosylation (CDG)

**DOI:** 10.1002/jgc4.1856

**Published:** 2024-01-19

**Authors:** Tara Weixel, Lynne Wolfe, Ellen F. Macnamara

**Affiliations:** ^1^ Department of Psychological Sciences Kent State University Kent Ohio USA; ^2^ Undiagnosed Diseases Program, National Human Genome Research Institute National Institutes of Health Bethesda Maryland USA

**Keywords:** congenital disorders of glycosylation, genetic counseling, genetic sequencing, inheritance, pediatrics, rare diseases

## Abstract

Congenital disorders of glycosylation (CDGs) are a genetically and clinically diverse group of disorders that arise as a result of defects within glycosylation synthetic pathways. CDGs are caused by pathogenic variants in many different genes in the glycosylation network. With over 160 different CDG types currently identified and a vast range of severity and presentations existing within and across those types, the road to a CDG diagnosis is often lengthy and complicated. The perils of this arduous CDG diagnostic odyssey are fraught with various genetic counseling uncertainties: (1) confusion about family planning, (2) queries about inheritance, (3) managing treatment, and (4) dealing with the uncertainty of rare diseases. Thus, the role of the genetic counselor is paramount in helping affected individuals and their families navigate these genetic counseling complexities. Case examples of common genetic counseling difficulties for CDGs are outlined, providing clinical applications of what CDG presentations, diagnostic processes, and common difficulties look like. Information on the nomenclature, incidence, prevalence, diagnostic testing, treatment, and management of CDGs are also discussed to provide a comprehensive summary of CDGs for genetic counselors, and subsequently to affected individuals and their families.

**What is known about this topic**

Congenital disorders of glycosylation (CDGs) are a diverse group of rare disorders that arise because of defects within glycosylation synthetic pathways with a vast range of presentation.

**What this paper adds to the topic**

Many difficulties transpire during the diagnostic odyssey of discovering individuals affected by CDGs. This paper outlines case examples of various CDG subtypes and inheritance patterns to provide genetic counselors with a comprehensive summary of how to provide support and care for affected individuals and their families.

## 1 INTRODUCTION

Congenital disorders of glycosylation (CDGs) are a genetically and clinically diverse group of rare inherited disorders that arise as a result of defects within the synthetic pathways of various glycoproteins and glycoconjugates. Currently, there are over 160 different CDG types identified (Boyer et al., 2022; Chang et al., 2018; Cylwik et al., 2013; Ng & Freeze, 2018; Ondruskova et al., 2021; Wilson & Matthijs, 2021).

Previously, CDGs had the nomenclature of “carbohydrate‐deficient glycoprotein syndromes”, but this term was updated to “congenital disorders of glycosylation” as the continued diversity of CDGs expanded (Aebi et al., 1999). Initially, CDG types were characterized as “Type I CDG” or “Type II CDG,” which were assigned according to the pattern of transferrin glycosylation isoform analysis (Jaeken et al., 2008). The pattern found in Type I CDGs results from dolichol‐linked glycan assembly and transfer defects localizing to the cytoplasm or endoplasmic reticulum. The pattern found in Type II CDGs results from processing defects in the Golgi apparatus. After a CDG type was assigned as Type I or Type II, the CDG type was then alphabetically assigned a letter in order of discovery. Therefore, the previous nomenclature used to name and identify CDG types consisted of the following format: (1) stating “CDG‐”, then (2) denoting the assigned type with “I” or “II,” which corresponds to “Type I" or “Type II," respectively, then lastly (3) assigning a letter in the alphabet to the new type in order of discovery. Following these steps, the most common and first CDG type ever discovered was named “CDG‐Ia” (now referred to as “PMM2‐CDG”). The “I” signified it was identified as a “Type I" pattern and the “a” signified it was the first discovered “Type I" CDG type.

Current naming convention lists the specific gene affected within the pathway and then “‐CDG” (Jaeken et al., 2009). Thus, CDG‐Ia becomes PMM2‐CDG as it is caused by pathogenic variants in phohphomannomutase (*PMM2*). The change in convention allows for easier recall and recognition of CDGs and for CDGs that do not follow a classic Type I or Type II transferrin pattern.

CDGs result from a perturbation in one of the synthetic glycosylation pathways including N‐linked glycosylation, O‐linked glycosylation, combined N‐ and O‐linked glycosylation, and lipid and glycosylphosphatidylinosital (GPI) anchor biosynthesis defects (Chang et al., 2018; Ng & Freeze, 2018). CDG types resulting from these genetic defects within glycosylation gene networks are considered primary CDGs. The diagnosis of a secondary CDG is made because of the presence of glycosylation abnormalities not caused by a defect in a synthetic glycosylation pathway, rather the abnormality is sequela from a different condition. An example of a secondary CDG is hereditary fructose intolerance (HFI), a metabolic disease caused by defective fructose metabolism. Along with the other symptoms of this disorder individuals with HFI may also have altered transferrin glycosylation patterns, a finding emblematic of CDGs (Quintana et al., 2009). Secondary CDGs can be misdiagnosed as primary CDGs and vice versa, and thus, proper diagnostic testing is essential when determining the diagnosis of CDG in affected individuals. Of note, there also exists one congenital disorder of *de*glycosylation, NGLY1‐CDDG, which is the result of a genetic defect of the *NGLY1* gene which codes for a protein catalyzing a deglycosylation step in the N‐linked synthetic pathway. Thus, it is often conceptually grouped with primary CDGs (Lam et al., 2017; Need et al., 2012).

Because there are over 160 CDG types, and differences in severity and presentation within those types, the typical features associated with CDGs vary widely both within and between types. Overall, CDGs are usually multi‐systematic and often present with developmental delay, failure to thrive, hypotonia, neurologic abnormalities, as well as varying abnormalities of the endocrinologic, hepatic, ophthalmologic, dermatologic, gastrologic, immunologic, skeletal, and coagulation systems. Not all CDG presentations are severe, some individuals with CDGs may present with only a single system affected (Chang et al., 2018; Gilfix, 2019). For this reason, CDGs should routinely be on the differential, especially for individuals with a multi‐systemic disease of an unknown etiology with neurologic abnormalities or developmental delay.

## 2 INCIDENCE AND PREVALENCE

The total incidence and prevalence for all CDG types is not known; however, cases have been documented worldwide, encompassing many ethnic backgrounds with both sexes affected. To date, most CDG types have 100 cases or fewer reported (Chang et al., 2018). For European populations and African American populations, the current estimated prevalence is 1/10,000 (Chang et al., 2018; Jaeken & Matthijs, 2007). For PMM2‐CDG, the most common type reported, at least 1000 cases have been documented worldwide, with the current estimated prevalence ranging from 1/20,000 in Dutch populations to 1/77,000 in Estonia (as per isolated reports) to 1/286,726 in Turkey (Chang et al., 2018; Schollen et al., 2000; Vals et al., 2018; Yıldız et al., 2020).

## 3 DIAGNOSTIC TESTING

An early and accurate diagnosis of a CDG allows for the timely implementation of appropriate management, improving the clinical outcomes for people with CDG. Diagnostic algorithms for CDGs have been proposed and a flowchart exists to aid in the implementation and interpretation of testing and results (Abu Bakar et al., 2018; Francisco et al., 2019; Lipiński & Tylki‐Szymańska, 2021). When CDGs were first identified, the first line of diagnostic testing was serum carbohydrate‐deficient transferrin (CDT) analysis. However, mass spectrometry, high‐performance liquid chromatography (HPLC), and capillary electrophoresis (CE)‐based methods have largely replaced CDT analysis as it was limited in its detection abilities to only N‐glycosylation defects with sialic acid deficiencies (Chang et al., 2018; Chen et al., 2019; Lefeber et al., 2011; Ng & Freeze, 2018; Park & Marquardt, 2021; Wada, 2020). With positive biochemical testing, genetic sequencing can then be used to identify the specific genetic defect. Consideration of the limitations of biochemical testing is important as some CDG types are not detectable by current testing strategies and may require the use of other diagnostic means (Witters et al., 2021). For CDG types with defects in N‐linked glycan synthesis, further diagnostic testing consists of enzyme assays, but notably, for many CDG types no enzyme assays are available (NORD, 2015; Van Scherpenzeel et al., 2016). In cases where there is a known family history of an individual with a CDG, then targeted gene or variant sequencing or a CDG gene panel may be possible (Jones et al., 2013).

With the advancement in genetic sequencing, a vast majority of individuals with CDGs are now diagnosed via genetic sequencing and confirmed with biochemical testing (Jones et al., 2013; Ng & Freeze, 2018). Exome and genome sequencing have led to the rapid discovery of new pathogenic variants related to known CDG types and the discovery of entirely new CDG types all together (Ng & Freeze, 2018). The use of sequencing is a particularly helpful diagnostic tool for CDG types given the limitations of biochemical testing.

## 4 FAMILY HISTORY RISK ASSESSMENT

The collection and use of a thorough family history provide a diagnostic tool and risk assessment for the presence of CDG types within families. Detailed pedigrees allow for ascertainment of symptoms that would otherwise seem unrelated to become connected, which is especially important given that CDG symptoms and presentations vary widely.

Since many, but not all CDGs are recessive in inheritance, the proband, and possibly their siblings, are typically the only affected individuals in their family. CDG carriers are currently not known to have symptoms. The knowledge and application of inheritance patterns for different CDG types further informs and guides diagnostic testing for CDGs. Therefore, recognizing the different inheritance patterns is vital for clinicians who are evaluating patients with known or suspected CDGs.

Ascertaining a thorough family history is a universally applicable genetic counseling tool. Although there are no specific questions that could be asked of all CDG patients and families, gathering family history in rare diseases can elucidate patterns not yet understood; the discovery of Parkinson's risk in Gaucher disease, is one example of this (Goker‐Alpan et al., 2004). Two examples of relevant family history in CDGs include the presence of multiple orthopedic surgeries in family members with EXT1‐CDG and the presence of lymphomas in maternal male relatives with MAGT1‐CDG (Ravell et al., 2014).

In addition to understanding and assessing family history and risk for CDGs, genetic counselors are vital for addressing other genetic counseling difficulties pertaining to CDGs. Examples of relevant genetic counseling difficulties for CDGs include understanding recurrence risk for future pregnancies, complicated inheritance (e.g., mosaicism and X‐linked considerations), managing and treating CDGs, and the uncertainty of navigating rare diseases. To provide comprehensive narratives and clear clinical applications of genetic counseling for CDGs, we have outlined the inheritance patterns and common genetic counseling difficulties in CDGs in the following sections via case examples. For case example data, the study was reviewed by the National Human Genome Research Institute's institutional review board and approved as human subjects research (HG000215‐20).

## 5 CASE EXAMPLE 1: PMM2‐CDG

A 3‐year‐old female and her 2‐year‐old sister present to a genetics clinic with developmental delay. The proband has a history of global developmental delay, failure to thrive, tonic–clonic seizures, suprapubic fat pad abnormalities, bilateral hypertropia of right eye with astigmatism, hypothyroidism, osteopenia, ventricular septal defect, and deficiencies of protein S, antithrombin III, factor IX and XI. The younger sister has a history of global developmental delay, hypotonia, failure to thrive, seizures, suprapubic fat pad abnormalities, gastrostomy tube placement, coagulopathy, and elevated liver function tests.

The initial diagnosis for these sisters was made by a geneticist who observed the developmental delay, multi‐systemic involvement, and suprapubic fat pad abnormality, which is characteristic of PMM2‐CDG (Monin et al., 2014; Sparks & Krasnewich, 2017). Subsequent genetic testing confirmed a diagnosis of PMM2‐CDG.

### 5.1 Counseling concern: Recurrence risk for future pregnancies

PMM2‐CDG is inherited in an autosomal recessive pattern. In the family described above, both parents were confirmed carriers of PMM2‐CDG. The recurrence risk for recessive conditions is quoted as one in four (25 percent). Theoretically, there is a one in two chance (50 percent) of having a child who is a carrier and a one in four chance (25 percent) chance of having an unaffected, noncarrier child.

However, Schollen analyzed 92 pregnancies of PMM2 families and found an increased recurrence risk due to transmission ratio distortion (Schollen et al., 2004). While the exact mechanism for this increased risk is unknown, it is hypothesized that the carrier sperm may be faster or perhaps the carrier eggs are easier to penetrate by sperm resulting in a higher chance of fertilization. Thus, many CDG families, even those with a different type of CDG, are told that there is an increased risk of a second, or another, affected pregnancy. Families report being told that the chance of having a second affected child is 33 percent or higher; one in three is the recurrence risk calculated by Schollen et al. They also report being told that the rate of miscarriage is increased in CDG families and that past miscarriages were the result of being CDG carriers. This is an important area for continued research; however, it is an understandably difficult dataset to gather and requires the collection of numerous cases throughout their reproductive journey. It is a good reminder of the benefit of centralized research efforts and the importance of referring families to the larger communities upon receipt of a diagnosis.

Knowing the correct recurrence risk for a condition is an important part of educational counseling; however, understanding how families and patients hear and interpret that information is critical. This is especially important when the data is different than the “general understanding.” The increased risk is true for some members of the CDG community and families have reported making reproductive decisions based on this chance of recurrence. As genetic counselors, we must know the recurrence risk, but it is crucial to understand how families hear and process the information they are told—the number is only as helpful as it is understood. Counselors should explore how families are using the information they have been given to make decisions and whether it is the number or the “feel” of the risk that is guiding these decisions.

## 6 CASE EXAMPLE 2A: EXT1‐CDG

An 8‐year‐old male presents to genetics clinic with multiple skeletal findings and normal growth and development. A skeletal survey shows coxa valga, osteochondromas of proximal humeral metadiaphyses, bilateral foreshortening of ulna, exostoses on femurs, tibial metaphases, fibula, and metatarsals.

An initial diagnosis was made via targeted EXT1 sequencing, which identified a de novo pathogenic variant, confirming a diagnosis of hereditary multiple osteochondromas, or EXT1‐CDG.

## 7 CASE EXAMPLE 2B: SLC35A2‐CDG

A 3‐year‐old female presented to genetics clinic with global developmental delay, seizures, cortical visual impairment, strabismus, failure to thrive, and leg length discrepancy. She has a history of recurrent vomiting and constipation leading to pyloric stenosis surgery at 9 weeks of age.

The initial diagnosis was made via trio genome sequencing, which revealed a de novo pathogenic mosaic variant in the Golgi SLC35A2 galactose transporter, indicative of SLC35A2‐CDG.

It is important to note that when conducting diagnostic screening via transferrin glycosylation, previous cohorts of individuals with SLC35A2‐CDG found a vast majority of them had normal transferrin glycosylation; however, primary fibroblasts allow for biochemical assays to be used to assess SLC35A2‐dependent UDP‐galactose transport activity (with previous research demonstrating transport activity is directly correlated to the ratio of wild‐type to pathogenic variants for individuals with SLC35A2‐CDG; Ng et al., 2019).

### 7.1 Counseling concern: Complicated inheritance, mosaicism, and X‐linked considerations

As the majority of CDGs involve severe developmental delay and medical concerns, affected individuals do not typically consider reproduction. However, there are CDGs, like EXT1‐CDG, where affected individuals having children should be discussed. EXT1‐CDG is an autosomal dominant condition and can be passed down to future generations; there is a 50 percent (one in two) chance that an affected individual will have an affected child (Delgado et al., 2014).

CDGs follow typical patterns of inheritance: autosomal dominant, recessive, and X‐linked dominant and recessive patterns have all been reported. For most recessive CDGs, heterozygotes are not known to have symptoms or health concerns related to their heterozygous variant. However, as research continues, we are learning that inheritance can be complicated. Francisco et al. (2019) for example, documented that EXT2‐CDG is a multiple exostoses syndrome with heterozygous variants, but presents with seizures, scoliosis, and macrocephaly as a biallelic condition. It is important to be aware of these complex inheritance patterns when providing recurrence risk and reproductive counseling to families.

Mosaic variants are also found in CDGs. Case Example 2B, for example, describes a case of a young girl who was found to have a mosaic variant in *SLC35A2*. Somatic mosaicism occurs when only a portion of cells are found to have the pathogenic variant, while the remaining cells do not have the pathogenic variant. In other words, some of the cells still work properly because they have an unaffected copy of *SLC35A2*.

SLC35A2‐CDG is an X‐linked dominant condition, which adds further considerations. The majority of reported cases are in affected females; males present with a more severe phenotype, causing a higher mortality rate for affected males as compared to affected females. It is hypothesized that a single damaged copy of *SLC25A2* is incompatible with life, as affected males all have mosaic variants (Ng et al., 2013).

## 8 CASE EXAMPLE 3: ALG13‐CDG

A 7‐year‐old female presents to genetics clinic with severe global developmental delay, epilepsy, autonomic dysfunction, cortical visual impairment, G‐tube dependency, eczema, allergic rhinitis, and latex allergy. She has a history of infantile spasms, failure to thrive, gastroesophageal reflux, apnea, and angioneurotic edema.

The initial diagnosis was made via genome sequencing, which revealed a likely pathogenic variant in the X‐linked gene *ALG13*, indicative of ALG13‐CDG.

### 8.1 Counseling concern: Management and treatment

Unfortunately, there are no therapeutic options for most types of CDGs and clinical management involves symptomatic care by a multi‐disciplinary team. The most common CDG, PMM2‐CDG, has international clinical guidelines that outline the recommendations for treatment (Altassan et al., 2019). Affected individuals are recommended to undergo routine evaluations, such as eye exams, hearing evaluations, and echocardiograms. Neuropsychological testing and physical, speech, and occupational therapy evaluations are essential as they can guide recommendations for Early Intervention, Individualized Educational Programs, alternative communication devices, and mobility equipment. Coordinated management by a multi‐disciplinary team improves medical outcomes and quality of life and comforts families who are caring for their children.

For a select few CDG types, targeted treatments are available to improve prognosis (Boyer et al., 2022; Joshi et al., 2016; Park & Marquardt, 2021; Verheijen et al., 2020; Witters et al., 2020). For example, oral mannose given to individuals with PMI‐CDG decreases their protein‐losing enteropathy, optimizes growth, and improves their quality of life. The clinical phenotype varies in ALG13‐CDG, but typically consists of developmental delay, intellectual disability, and epileptic encephalopathy (Alsharhan et al., 2021; Galama et al., 2018). In ALG13‐CDG, ketogenic diets have been suggested as an effective seizure management option (Ng et al., 2020). Although there are no current cures, there is reason to be hopeful for better treatments for CDGs in the future. There are many robust CDG support communities, including the CDG Global Alliance, CDG CARE, and other international CDG‐type‐specific groups. These groups work to fundraise, connect patients with clinicians, and push research forward. Research on other possible treatments, such as gene therapy, continue to be conducted, with more possible treatments available in the future (Haenseler et al., 2018; Verheijen et al., 2020).

## 9 CASE EXAMPLE 4: PIGA‐CDG

A 4‐year‐old male and his 2‐year‐old brother present to a neurology clinic with a history of seizures. The proband has Generalized Epilepsy with Febrile Seizures Plus (GEFS+). He also has a history of ear infections, adenoid hypertrophy, for which he had an adenoidectomy and nasal airway obstruction. The younger brother has a history of febrile generalized tonic–clonic seizures, otorrhea, dysfunction of the eustachian tube, chronic otitis media—for which bilateral myringotomies with tube placement were performed, and eczema.

The initial diagnosis for the proband was made via EpiXpanded Panel, which conducted a sequence analysis of 1386 genes and found pathogenic variants of the *PIGA* gene, indicative of PIGA‐CDG. Receipt of this genetic diagnosis for the proband led to the young brother receiving a PIGA‐CDG diagnosis through familial variant testing.

### 9.1 Counseling concern: Uncertainty in rare diseases

Many families search for a diagnosis in hopes of reducing uncertainty (McKoane & Sherman, 2022). But uncertainty remains even when a diagnosis is given, and especially when the diagnosis is a rare disease for which much remains unknown about treatment and prognosis. This uncertainty can be more acutely felt when two siblings have the same condition with varying presentations—trying to gauge whether the severity of their disease will be the same is difficult. It is important to reinforce that even with a diagnosis, children are allowed to be individuals. They may differ from other children with the same condition, and they may even differ from their siblings with the same condition. Once a diagnosis is known, in rare diseases, it is the child that will continue to teach us what the course of disease will look like for them.

It may be helpful to provide this counseling as part of anticipatory guidance. Things can change when a diagnosis is known—a more specific prognosis, recommendations for management, and accurate recurrence risk, but nothing has changed about the child receiving the diagnosis. The child is still the same person, their parents are still the experts in what their disease looks like in them, and the child will chart their own course.

## 10 CONCLUSION

Whenever an individual presents with a multi‐systemic disease of an unknown etiology, particularly an unknown multi‐systemic disease with neurologic abnormalities or developmental delay, the diagnosis of CDG should be considered. As the number of recognized types of CDGs continues to expand, the uncertainty encompassing the diagnostic process for CDG diagnoses continues to challenge health care providers, affected individuals, and their families. The complexity of the clinical and genetic presentations for people with CDGs present significant challenges for health care providers, patients, and their families. These case presentations highlight the optimal approach to diagnose, counsel, and care for patients with suspected or known CDGs. Many affected individuals and their families navigate confusion about family planning, queries about inheritance, managing treatment, and dealing with the uncertainty of rare diseases. Thus, the role of the genetic counselor is paramount in helping affected individuals and their families navigate these genetic counseling complexities.

## AUTHOR CONTRIBUTIONS

Tara Weixel and Ellen Macnamara contributed substantially to the conception of the work, the acquisition, analysis, and interpretation of data for the work, drafting the work, revising the work critically for important intellectual content, and final approval of the version to be published. Lynne Wolfe contributed to revising the work critically for important intellectual content and final approval of the version to be published. Tara Weixel, Lynne Wolfe, and Ellen Macnamara confirm that they had full access to all the data in the study and take responsibility for the integrity of the data and the accuracy of the data analysis. All of the authors gave final approval of this version to be published and agreed to be accountable for all aspects of the work in ensuring that questions related to the accuracy or integrity of any part of the work are appropriately investigated and resolved.

## CONFLICT OF INTEREST STATEMENT

Tara Weixel, Lynne Wolfe, and Ellen Macnamara declare that they have no conflict of interest.

## ETHICS STATEMENT

Human Studies and Informed Consent: Approval to conduct this human subjects research was obtained by the National Human Genome Research Institute's institutional review board. All procedures followed were in accordance with the ethical standards of the responsible committee on human experimentation at the National Human Genome Research Institute and with the Helsinki Declaration of 1975, as revised in 2000. Informed consent was obtained from all patients/guardians for being included in the study.

Animal Studies: No non‐human animal studies were carried out by the authors for this article.

## Data Availability

The data that support the findings of this study are available from the corresponding author upon reasonable request.
